# Research on the Efficiency of Bridge Crack Detection by Coupling Deep Learning Frameworks with Convolutional Neural Networks

**DOI:** 10.3390/s23167272

**Published:** 2023-08-19

**Authors:** Kaifeng Ma, Xiang Meng, Mengshu Hao, Guiping Huang, Qingfeng Hu, Peipei He

**Affiliations:** College of Surveying and Geo-Informatics, North China University of Water Resources and Electric Power, Zhengzhou 450046, China; z20201150991@stu.ncwu.edu.cn (X.M.); hm3108197908@outlook.com (M.H.); huangguiping123@163.com (G.H.); huqingfeng@ncwu.edu.cn (Q.H.); hepei@ncwu.edu.cn (P.H.)

**Keywords:** DLF, object detection model, semantic segmentation model, bridge crack detection, evaluation indicators

## Abstract

Bridge crack detection based on deep learning is a research area of great interest and difficulty in the field of bridge health detection. This study aimed to investigate the effectiveness of coupling a deep learning framework (DLF) with a convolutional neural network (CNN) for bridge crack detection. A dataset consisting of 2068 bridge crack images was randomly split into training, verification, and testing sets with a ratio of 8:1:1, respectively. Several CNN models, including Faster R-CNN, Single Shot MultiBox Detector (SSD), You Only Look Once (YOLO)-v5(x), U-Net, and Pyramid Scene Parsing Network (PSPNet), were used to conduct experiments using the PyTorch, TensorFlow2, and Keras frameworks. The experimental results show that the Harmonic Mean (F1) values of the detection results of the Faster R-CNN and SSD models under the Keras framework are relatively large (0.76 and 0.67, respectively, in the object detection model). The YOLO-v5(x) model of the TensorFlow2 framework achieved the highest F1 value of 0.67. In semantic segmentation models, the U-Net model achieved the highest detection result accuracy (AC) value of 98.37% under the PyTorch framework. The PSPNet model achieved the highest AC value of 97.86% under the TensorFlow2 framework. These experimental results provide optimal coupling efficiency parameters of a DLF and CNN for bridge crack detection. A more accurate and efficient DLF and CNN model for bridge crack detection has been obtained, which has significant practical application value.

## 1. Introduction

**Figure 1:**
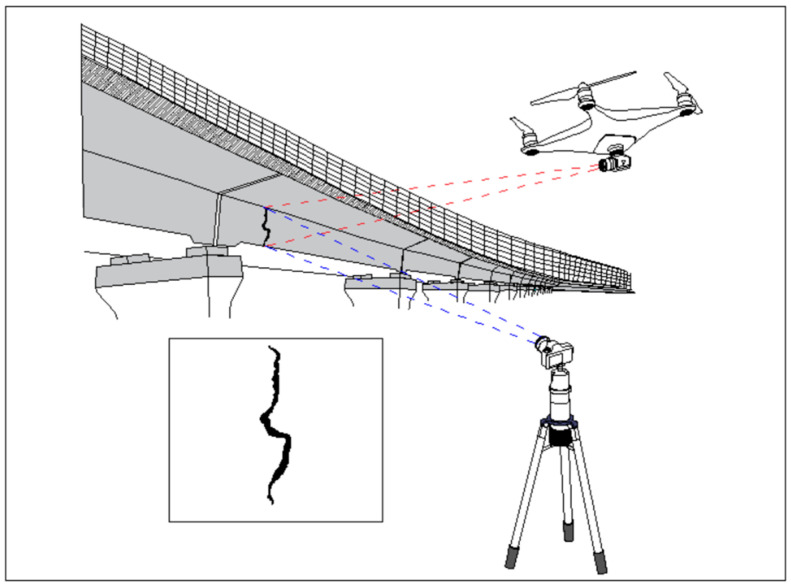
Bridge cracks detection.

With the rapid economic development in China, the construction of transportation infrastructure has advanced significantly, and the field of bridge construction has achieved a world-leading position. According to the 2021 statistical bulletin on the development of the transportation industry from the Ministry of Transport, there were 961,100 highway bridges nationwide, with a total length (TL) of 73,802,100 m. This represents an increase of 48,400 bridges, with a TL of 7,516,600 m, compared to the end of the previous year, of which 7417 were super-large bridges with a TL of 13,478,700 m, and 134,500 were medium and small bridges with a TL of 37,158,900 m. Therefore, it can be seen that the current number of bridges in China is quite large. During the operation of the bridges, cracks are prone to occur due to excessive loads, poor-quality construction materials, heat, and other factors [1,2,3]. Cracking is a severe problem for bridges, as it can significantly affect their safe operation, resulting in economic losses and casualties once an accident occurs [4,5,6]. Therefore, the timely and accurate detection of bridge cracks has become a primary task of bridge maintenance [7]. Crack identification and positioning are important parts of bridge safety detection. At present, the detection method for bridge cracks in China is mainly manual detection. Inspection personnel generally use telescopes, ladders, bridge inspection vehicles, and other tools for manual measurement, analysis, and evaluation. Although manual detection is simple and flexible, it has several shortcomings, including low efficiency, poor accuracy, and high cost [8]. The digital image processing method based on grayscale edge extraction is commonly used for bridge crack detection. Unmanned Aerial Vehicles (UAVs), cameras, and other equipment are used to collect crack images for bridge cracks. Figure 1 shows the position and properties of bridge cracks that can be extracted [9,10,11,12]. Li et al. [13] detected cracks using the classical Sobel operator and connected domain measurement. Huang and Tsai [14] proposed a dynamic optimal threshold segmentation algorithm based on dynamic programming, and Kirschke and Velinsky [15] proposed a crack image thresholding algorithm based on sub-image grayscale histograms. Tsai et al. [16] used the maximum inter-class variance method for crack detection. However, due to the complex background of bridge crack images, the crack detection results often carry a large amount of noise, resulting in low detection accuracy.

In order to improve the efficiency and accuracy of bridge crack detection, several scholars have conducted research on the influence of background noise on recognition results [4,11,17,18,19]. To provide a more accurate and efficient DLF and CNN model for bridge crack detection, this paper experiments with different DLFs and CNNs, to evaluate their coupling effect on bridge crack detection efficiency. Deep learning techniques utilize CNN models for tasks such as object detection, semantic segmentation, and instance segmentation within a specific learning framework. Many scholars have studied crack detection using different neural network models based on existing learning frameworks, such as PyTorch, TensorFlow, and Keras. For instance, Amo-Boateng [20] utilized a Mask-RCNN model network to achieve a cross-merge ratio of 88.2% in house roof segmentation based on the TensorFlow framework. Ref. [21] used an improved Full Convolutional Neural Network (FCNN) model for crack detection, achieving an average cross-merge ratio of 55.2% for detection results. Fu et al. [22] applied the DeepLabv3+ model for crack segmentation and achieved an average cross-merge ratio of 82.37%. Based on the Keras framework, Zhang et al. [23] utilized the improved UNet model for crack segmentation to detect concrete cracks with high accuracy. Ochoa-Ruiz et al. [24] used the RetinaNet model for crack detection, achieving object detection with higher accuracy and efficiency. Qu and Xie [25] applied the full U-shaped network model for crack segmentation and achieved a 1.48% increase in *p*, a 4.68% increase in R, and a 3.29% increase in F values. Based on the PyTorch framework, Jia et al. [26] used the improved EfficientDet model to detect marine organisms with a detection rate of over 90%, while Yu et al. [27] used the CenterNet model for vehicle detection, with an average accuracy of 94.74%. Liu et al. [28] proposed the YOLOv3-FDL model with a four-scale detection layer. Compared with the original YOLOv3 model, the F1 score and mAP of the YOLOv3-FDL model reached 88.8% and 87.8%, respectively, which increased by 8.8% and 7.5% on the GPR data set, respectively. Wang et al. [29] combined data enhancement and structure optimization to significantly improve the accuracy of the YOLOv3 object detection model. Zhang et al. [30] utilized the improved YOLO v4 model for crack detection, achieving experimental results of 93.96%, 90.12%, and 92.00% for *p*, R, and F values, respectively. In order to reduce the interference of complex background factors of crack images on the crack detection results, Yu [31] adopted the threshold segmentation method based on the maximum inter-class variance of Otsu to preprocess images and conducted the crack detection task by using YOLOv5s, and achieved good results. Qiao et al. [32] utilized the improved U-net model for crack segmentation, resulting in an average accuracy 11.7% higher than the U-net neural network segmentation model.

In short, most scholars choose the DLF and CNN model to conduct corresponding research based on the existing foundation. It is not clear whether the chosen DLF and CNN model are the optimal combination. Moreover, no systematic experimental research has been found on the coupling efficiency of the DLF and CNN model. Based on this, this article aimed to investigate the effectiveness of coupling the DLF with the CNN model for bridge crack detection. To verify the effectiveness of coupling a DLF and CNN for bridge crack detection, bridge crack datasets were collected and labeled, and the DLF and CNN model was introduced to perform bridge crack detection experiments. The coupling efficiency of the DLF and CNN model was tested, and the optimal object detection and semantic segmentation CNN model for bridge crack detection under a DLF was obtained. This provides a practical basis for selecting the CNN model and DLF for efficient and high-precision bridge crack detection.

## 2. Methods

### 2.1. Deep Learning Framework (DLF)

#### 2.1.1. PyTorch

PyTorch is a DLF developed by Facebook. It uses Python as the primary programming language and is based on the original Torch framework. PyTorch has two distinguishing features: dynamic computational graphs and simplicity. With PyTorch, developers can easily modify and debug their code, making it more flexible than TensorFlow. The progression from data tensor to network abstraction levels is ‘Tensor to Variable to nn.Module’, respectively [33].

#### 2.1.2. TensorFlow

TensorFlow 1.0 uses static graphs that are difficult to debug, while TensorFlow 2.0 uses dynamic graphs that come with a debugging tool. TensorFlow has broader serialization and deployment support than PyTorch, making it less prone to flaws. TensorFlow can be used to implement other machine learning algorithms besides neural networks because its data flow graphs support very free algorithmic representations [33].

#### 2.1.3. Keras

Keras is a high-level DLF based on the underlying operations provided by frameworks such as Theano and TensorFlow. Keras is easy to use and can implement complex models with few lines of code. It specializes in deep learning, supporting CNNs, and recurrent networks. Keras can be accelerated by replacing the CPU with a GPU [33].

The differences among the characteristics of the three DLFs [33] are presented in Table 1.

### 2.2. CNN Models

#### 2.2.1. Object Detection Network Model

(1) Faster R-CNN model

Faster R-CNN, a typical representative of the two-stage object detection model, was proposed by Girshick et al. [34] in 2015. Based on Fast R-CNN, this network uses a small region proposal network (RPN) instead of a selective search algorithm, which greatly reduces the number of candidate boxes and thus greatly improves the detection speed of object detection [35]. To detect cracks, we employed the Faster R-CNN model, which leverages the Resnet 50 backbone feature extraction network. The Resnet 50 network comprises a preprocessing module and four convolutional modules. The preprocessing module contains a convolutional layer and a pooling layer. Specifically, the convolutional layer uses a 7 × 7 kernel size with a stride of 2, whereas the pooling layer adopts the Max Pooling method with a 3 × 3 kernel size and a stride of 2, and the activation function is set as the ReLU function, as shown in Formula (1). These operations enable the Resnet 50 network to obtain a common feature layer for crack detection.

(1) $$y=\left\{\begin{array}{c} x\;(x>0)\\ 0\;(x\leq 0) \end{array}\right.$$

**Figure 2:**
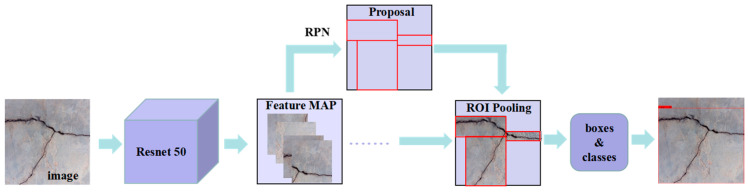
Faster R-CNN model.

The Resnet 50 network takes an input image size of (600, 600, 3). The image size is then converted through the process, successively starting from (600, 600, 3) and proceeding to (300, 300, 64), (150, 150, 64), (150, 150, 256), and (75, 75, 512), and finally obtaining a shared feature layer of (38, 38, 1024), using the Resnet 50 backbone feature extraction network. A Region Proposal Network (RPN) generates a base a priori frame, with a 3 × 3 convolution used to integrate features. The prior frame is adjusted through regression prediction by adjusting four parameters: width, height, and central coordinates x and y. The training parameters are frozen at 10^−4^ and unfrozen at 10^−5^. The adjusted a priori frame is transformed into a proposal frame, which is then used to intercept the shared feature layer. Finally, classification and regression predictions are performed based on the prediction results, and the specific process is demonstrated in Figure 2.

(2) SSD model

**Figure 3:**
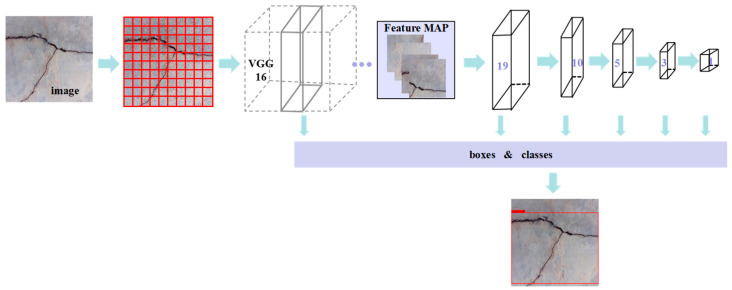
SSD model.

SSD is a single-stage object detection algorithm proposed by Liu et al. [36] in ECCV, 2016. This algorithm combines the regression idea in YOLO with the Anchor mechanism in Faster-RCNN, and uses multi-scale regions of each position in the whole map for regression, which can improve the running speed and ensure detection accuracy [37]. Crack detection based on the SSD model involves dividing the original input image into M×N grids, generating n prior frames for each grid, with the anchor sizes of the prior frames being (30, 60, 111, 162, 213, 264, and 315). For backbone feature extraction, the VGG 16 network is utilized, with an input image size of (600, 600, 3), and the activation function is set as the ReLU function. A convolution step of 2 and the Max Pooling method are used for pooling. The image size is then converted through the process, starting from (300, 300, 3) and proceeding to (300, 300, 64), (150, 150, 128), (75, 75, 256), (38, 38, 512), and finally (19, 19, 1024). The prior frame is then adjusted by modifying four parameters, namely width, height, and central coordinates x and y. This process generates a prediction frame, with the model being trained with a maximum learning rate of 0.002 and a minimum learning rate of 0.00002. Regression analysis and classification prediction are performed on each prediction frame, and the results are then integrated to produce the final prediction results; the specific process is demonstrated in Figure 3.

(3) YOLO-v5(x) model

**Figure 4:**
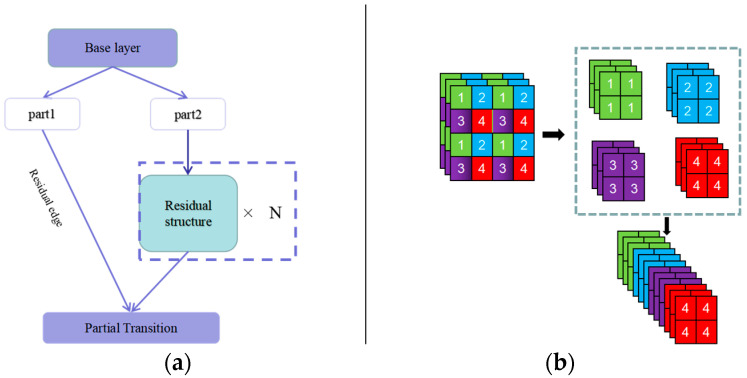
Network model structure. (**a**) CSPDarknet53 network; (**b**) Focus network.

YOLOv5 is a single-stage object detection algorithm released by the Ultralytics company in 2020. It was improved on the basis of the YOLOv3 model and belongs to the extension of the YOLO series [38]. The YOLOv5 model consists of a backbone, neck, and head. The YOLO-v5(x) model adopted in this paper is the largest model in the YOLOv5 series, with more layers and higher computational complexity, as well as the most powerful detection performance [39]. In crack detection based on the YOLO-v5(x) model, the original input map is divided into M×N grids with n prior frames generated for each grid. The anchors_mask of prior frames is ((6, 7, 8), (3, 4, 5), and (0, 1, 2)). The CSPDarknet53 network (Figure 4a) and Focus network (Figure 4b) are used for the extraction of backbone features. The CSPDarknet53 network uses a residual network structure for convolution, which links the feature extraction results to the original image and reduces the occurrence of missed and false detections during the detection process. The Focus network obtains one value per pixel in an image and four separate layers.

The entered image size is (640, 640, 3) and the channels are converted from 3 to 64. The SPP model structure is introduced as an enhanced feature extraction network, and pooling kernels of 5, 9, and 13 in size are used for Max Pooling. The convolution step is 1, and the activation function is a Sigmoid function, as shown in Formula (2):

(2) $$y=\frac{1}{1+e^{-z}}$$

**Figure 5:**
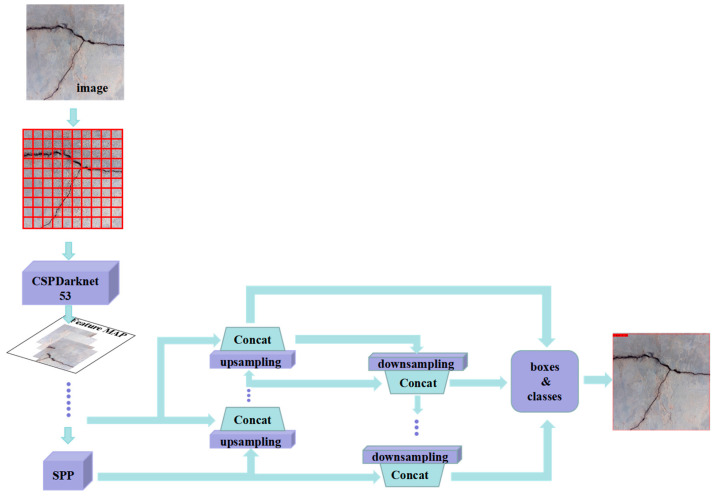
YOLO-v5(x) model.

The obtained features are up-sampled using the PANet network and further extracted. The same size features obtained by down-sampling and up-sampling are stacked, and the prior frame is adjusted to generate prediction frames. Regression analysis and classification prediction are performed for each prediction frame, and all the results are integrated to produce the final prediction results. In this paper, the YOLO-v5(x) version is used, and the specific process is demonstrated in Figure 5.

#### 2.2.2. Semantic Segmentation Network Model

(1) U-Net model

**Figure 6:**
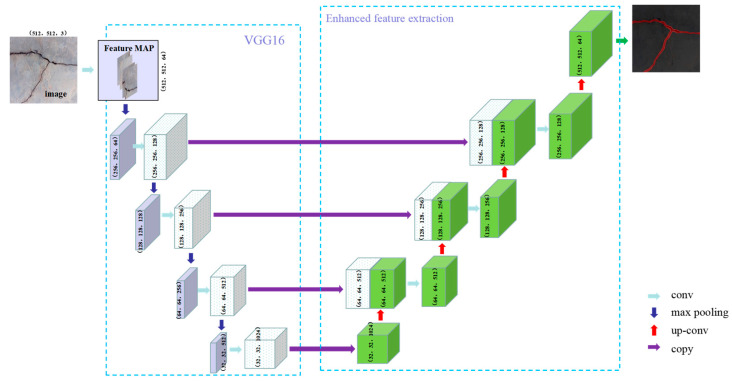
U-Net model.

U-Net is a classic codec network, which was proposed by Ronneberger et al. [40] in 2015 and was designed primarily for medical image segmentation. The core idea of this network is to introduce skip connection, use feature splicing for feature fusion, and make full use of image context information, which greatly improves the accuracy of image segmentation, and is almost the most widely used model in current semantic segmentation projects [23]. Based on the U-Net model for crack segmentation, the input image size is (512, 512, 3). The ReLU function is used as the activation function, while Max Pooling is utilized for the pooling method, and the convolutional step is 2. The U-Net model is divided into three parts: backbone feature extraction, enhanced feature extraction, and model segmentation prediction. For the backbone feature extraction model, VGG16 is employed to obtain five initial effective feature layers, successively starting from (512, 512, 64) and proceeding to (256, 256, 128), (128, 128, 256), (64, 64, 512), and finally (32, 32, 512). The enhanced feature extraction up-samples the five effective feature layers above, starting from (32, 32, 512) and proceeding to (64, 64, 512), (128, 128, 256), (256, 256, 128), and finally (512, 512, 64). The feature stacks the results of backbone feature extraction with the same size and channel and the enhanced feature extraction to obtain the final effective feature layer. The maximum learning rate of the model is set to 10^−4^, while the minimum learning rate is set to 10^−6^. The model segmentation prediction classifies each feature point on the final effective feature layer to obtain the final prediction results. The specific process is illustrated in Figure 6.

(2) PSPNet model

**Figure 7:**
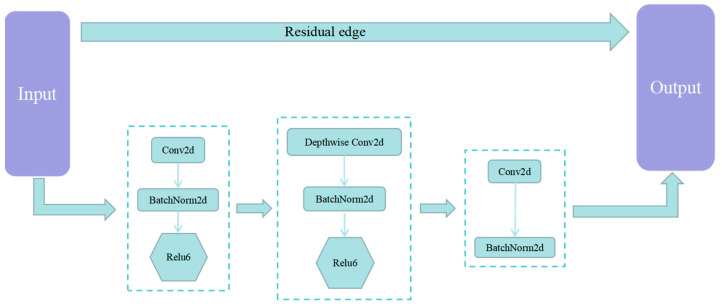
Mobilenet v2 network model structure.

PSPNet was proposed by Zhao et al. [41] in 2017, mainly to solve the problems existing in FCN networks for scene analysis tasks. The pyramid pooling module proposed by this model can aggregate context information of different regions, thereby improving the ability of the network model to obtain global information [18]. The PSPNet model is used for crack segmentation, and the input image size is (473, 473, 3). The ReLU function is used for the activation function, and the convolutional step is 2. The network is divided into two parts. The first part of the backbone feature extraction model uses a MobileNet v2 network. The network consists of three parts: ascending, depth-separable convolution, and descending, with the ascending and descending parts mainly changing their channels and the depth-separable convolution extracting the features from the changed channels, as shown in Figure 7.

**Figure 8:**
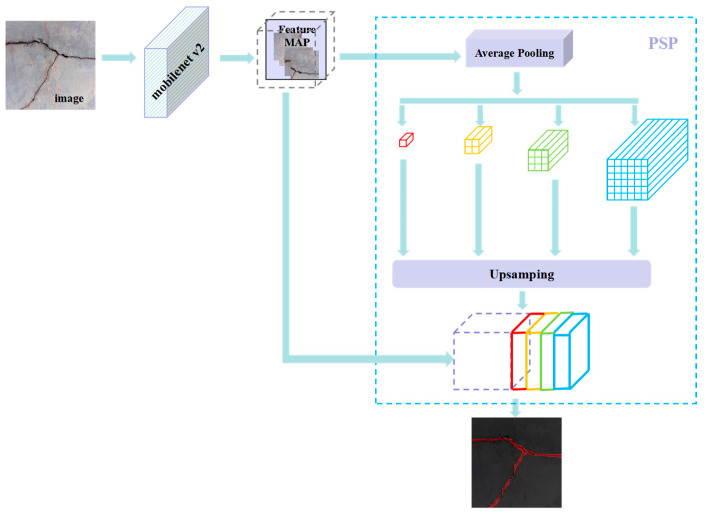
PSPNet model.

The PSP module is introduced to segment the acquired effective feature layer into 1 × 1, 2 × 2, 3 × 3, and 6 × 6 regions, using the Average Pooling method for each region with a convolution step of 1. The pooling result is up-sampled and stacked with the second part of the feature image, and the pixel points are finally classified, as shown in Figure 8.

## 3. Results

### 3.1. Experimental Data

The crack detection image dataset used in this paper is derived from reference [42]. The number of images is 2068, and the pixel size is 1024 × 1024 pixels, where the number ratio of the randomly selected training set, validation set, and testing set is 8:1:1, respectively. The processor used for model training is the Intel(R) Core(TM) i7-8700K CPU @ 3.70 GHz. The labelling tool used for object detection is ‘labelimg’ and the generated file is .xml for object detection, and LabelMe for semantic segmentation, generating a .json file. The configuration parameters of the deep learning framework are displayed in Table 2.

### 3.2. Experimental Results

**Figure 9:**
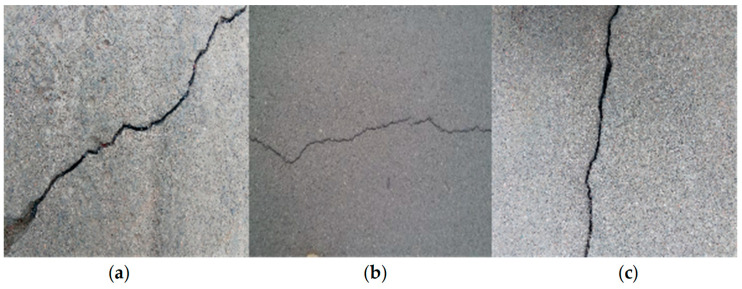
Original images used in the experiment. (**a**) Oblique cracks; (**b**) horizontal cracks; and (**c**) vertical cracks.

In the process of verifying the coupling efficiency of the model, three images of bridge cracks with different orientation characteristics were selected in the experimental data set in order to not lose generality. The directional characteristics of bridge cracks are inclined, horizontal, and vertical, respectively, as shown in Figure 9.

(1) Object detection

In order to test the coupling efficiency of different DLFs and CNN models in the identification and detection of bridge cracks, a bridge crack object detection experiment was carried out, and the results of bridge crack prediction were obtained. It can be seen that the different DLFs and CNN models could accurately detect bridge cracks and judge their effect of predicting bridge cracks, so as to calculate the confidence of their predicted crack results. Detection results for different DLFs and CNN models in object detection are as shown in Table 3.

As can be seen from the experimental results, the Faster R-CNN detection results with the lowest confidence root mean square (RMS) of 0, 0.015, and 0.006 in the three bridge crack detection images based on the PyTorch, TensorFlow2, and Keras frameworks, performed the best. Under the same conditions, the detection effect of the YOLO-v5(x) was second, and the detection effect of SSD was relatively worst. The prediction confidence for different DLFs and CNN models in object detection are displayed in Table 4.

**Figure 10:**
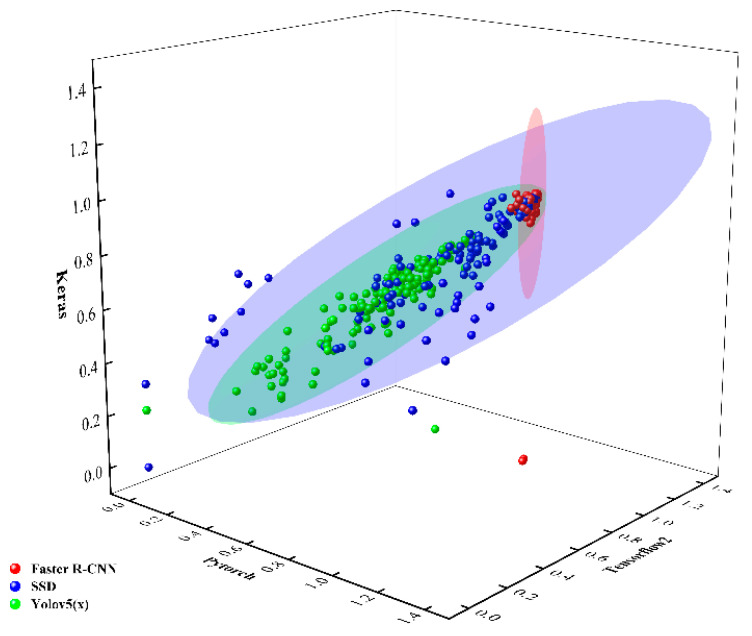
Prediction confidence of each network model under different DLFs.

To illustrate the dispersion of different CNN models under different DLFs, 131 images were randomly selected for confidence prediction analysis in this experiment. The confidence levels detected by the Faster R-CNN were more densely distributed in the PyTorch, TensorFlow2, and Keras frameworks. Conversely, the confidence levels detected by the SSD were more scattered in the PyTorch, TensorFlow2, and Keras frameworks. For the YOLO-v5(x) in PyTorch, TensorFlow2, and Keras, the confidence levels were generally lower and more scattered. The results are demonstrated in Figure 10.

In order to further investigate the average prediction level of different CNN models, the confidence was analyzed and processed. The confidence mean value and RMS results for different frameworks and networks in object detection were obtained. It can be observed that for the Faster R-CNN in the PyTorch, TensorFlow2, and Keras frameworks, the mean confidence values were 0.993, 0.978, and 0.964, respectively. The predictive confidence RMS values were 0.011, 0.019, and 0.122, respectively. The CNN model exhibits a small fluctuation range in PyTorch and TensorFlow2, and a relatively large fluctuation range in Keras. For the SSD in the PyTorch, TensorFlow2, and Keras frameworks, the mean confidence values were 0.782, 0.731, and 0.765, respectively. The predictive confidence RMS values were 0.275, 0.214, and 0.200, respectively. The CNN model displayed a moderate and similar range of fluctuations in the PyTorch, TensorFlow2, and Keras frameworks. Regarding YOLO-v5(x), the mean confidence values were 0.590, 0.592, and 0.598, with RMS values of 0.156, 0.161, and 0.152, in the PyTorch, TensorFlow2, and Keras frameworks, respectively. The three fluctuations were large and similar, and the overall confidence level was relatively low. The results are shown in Table 5.

(2) Semantic segmentation

To calculate the width and length of the detected cracks, experiments on the semantic segmentation of the cracks were performed, and the results of the segmented cracks were obtained, as shown in Table 6. Table 6 shows that, using the PyTorch framework, the U-Net was able to perform more comprehensive crack segmentation compared to the PSPNet, which was less sensitive to crack recognition and produced incomplete crack segmentation. When using the TensorFlow2 framework, both U-Net and PSPNet could segment cracks more comprehensively, meeting the requirements for crack segmentation. On the other hand, when using the Keras framework, the U-Net was less sensitive to crack recognition, while the PSPNet produced localized segmentation of cracks and fell short of the segmentation requirement.

### 3.3. Evaluation Indicators

**Figure 11:**
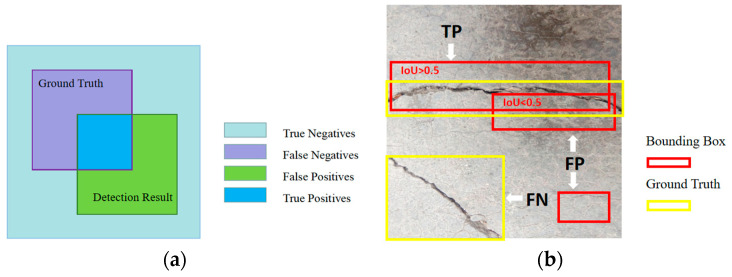
Relationships between parameters. (**a**) Semantic segmentation; (**b**) object detection.

To assess the performance of a network model in object recognition and classification, common evaluation metrics include the *p*-value (Precision), R-value (Recall), and summation mean (F1). These metrics were used to determine the effectiveness of the network model in recognizing and classifying objects. The evaluation indicators of semantic segmentation were usually calculated using true-positive (TP), true-negative (TN), false-positive (FP), and false-negative (FN) parameters, and the relationships between them are shown in Figure 11a. In the object detection evaluation metrics, TP means that the intersection over union (IoU) of the predicted bounding boxes and the ground truth (GT) is greater than the specified threshold. If there are multiple predicted bounding boxes for a GT and the IoU of the predicted bounding boxes are all greater than the specified threshold, the predicted bounding boxes of the largest IoU are TP and the excess TP is considered FP, i.e., only one TP is computed for a GT. FP means that the IoU of the predicted bounding boxes and the GT are less than or equal to the specified threshold or there are no predicted bounding boxes to be detected. FN means GT without TP. In this experiment, the IoU threshold was set to 0.5. The relationships are shown in Figure 11b.

(1) Precision values

The precision value represents the proportion of correctly retrieved results to all actually retrieved results and is calculated as follows:

(3) $$P=\frac{TP}{TP+FP}$$

(2) Recall values

The Recall value represents the proportion of positive class data actually predicted correctly to the results of all positive classes and is calculated as follows:

(4) $$R=\frac{TP}{TP+FN}$$

(3) Harmonized averages

The accuracy or recall alone is not a good indicator of a model’s performance. The summed average is a neutralizer of accuracy and recall.

(5) $$F1=\frac{2TP}{2TP+FP+FN}$$

(4) Accuracy

Accuracy reflects the proportion of all correct predictions (both positive and negative) to the total number of test results.

(6) $$AC=\frac{TP+TN}{TP+FP+TN+FN}$$

To characterize the effectiveness of the crack identification effect, the detection results of the testing dataset were tallied, and the *p*-value, R-value, and harmonic mean F1-value were obtained for this dataset. The CNN models designed for the same object detection task exhibited varying detection performance across three DLFs—PyTorch, TensorFlow2, and Keras. In Keras, the Faster R-CNN and SSD models yielded the highest *p*, R, and F1 values, with 80.53%, 71.37%, and 0.76, and 82.76%, 56.47%, and 0.67, respectively. Meanwhile, the YOLO-v5(x) model achieved the highest *p*-value of 88.28% in PyTorch and the highest R and F1 values in TensorFlow2, with 54.9% and 0.67, respectively. Across the same deep learning framework, different CNN models designed for object detection yield different detection outcomes. Specifically, the YOLO-v5(x) model achieved the highest *p*-values, whereas the Faster R-CNN model delivered the highest R and F1 values across all frameworks. Therefore, selecting the optimal CNN model for bridge crack detection in practical applications depends on the DLF used. The results are shown in Table 7.

Additionally, to evaluate the crack segmentation performance, the *p*-value, R-value, and AC-value were computed for the testing dataset. The performance of the same CNN model for semantic segmentation varied under different DLFs, namely, PyTorch, TensorFlow2, and Keras. Among the frameworks, the U-Net model exhibited the highest *p*-value and AC-value under PyTorch, with scores of 89.11% and 98.37%, respectively. The same model achieved the highest R-value under TensorFlow2, with a score of 92.38%. On the other hand, the PSPNet model achieved the highest *p*-value of 88.36% under Keras, and the highest R-value and AC-value of 87.86% and 97.86%, respectively, under TensorFlow2. It is worth noting that the performance of the different CNN models for semantic segmentation varied even when implemented under the same framework. For instance, the U-Net model achieved the highest *p*, R, and AC values under both PyTorch and TensorFlow2 frameworks, whereas the PSPNet model achieved the highest *p*, R, and AC values under the Keras framework. Therefore, the selection of the most suitable CNN model for bridge crack segmentation in practice should be based on the DLF used to achieve the best results. The results are presented in Table 8.

## 4. Discussion

Researchers such as Ji et al. [43] and Liu et al. [44] conducted related studies on object detection network models, utilizing the Faster R-CNN model, whereas Wan et al. [45] and Wang et al. [46] used the SSD model, and Yu et al. [47] and Li et al. [48] used the YOLO-v5 model, all of which achieved promising results. In this study, we have primarily focused on conducting coupled detection effectiveness experiments using the aforementioned three CNN models in conjunction with three DLFs. The most impressive results obtained include a *p*-value of 74.67% and an R-value of 67.06% for the Faster R-CNN model in TensorFlow2, a *p*-value of 79.35% and an R-value of 51.37% for the SSD model in TensorFlow2, and a *p*-value of 88.28% and an R-value of 50.2% for the detection results in YOLO-v5(x) using PyTorch. Regarding semantic segmentation network models, similar studies were conducted using the U-Net model [49,50], and using the PSPNet model [20,51], which also yielded improved results. However, this paper has mainly focused on conducting coupled detection performance experiments utilizing the two above-mentioned CNN models in conjunction with three DLFs. The best results obtained include an AC-value of 98.28% for the U-Net model in TensorFlow2 and an AC-value of 97.86% for the PSPNet model in TensorFlow2.

### 4.1. CNN Model with Different DLFs in the Same Dataset

Regarding object detection network models, the Faster R-CNN and SSD models showed the best overall performance in Keras, with F1 values of 0.76 and 0.67, respectively. By contrast, the YOLO-v5(x) model had a *p*-value of only 88.28% in PyTorch, but it performed the best overall in TensorFlow2, with R and F1 values of 54.9% and 0.67, respectively.

As for the semantic segmentation network models, the U-Net model had the best overall performance in PyTorch, with *p* and AC values of 89.11% and 98.37%, respectively, although its R-value was only 92.38% in TensorFlow2. On the other hand, the PSPNet model had a *p*-value of only 88.36% in Keras, but it achieved the best overall performance in TensorFlow2, with R and AC values of 87.86% and 97.86%, respectively.

### 4.2. Different CNN Models with the Same Dataset and the Same DLF

Among the object detection network models tested in PyTorch, TensorFlow2, and Keras, the YOLO-v5(x) model achieved the largest *p* values of 88.28%, 87.5%, and 84.71%, respectively, whereas the Faster R-CNN model achieved the largest R and F1 values (with R values reaching 64.71%, 67.06%, and 71.37%, and F1 values reaching 0.69, 0.71, and 0.76, respectively). By considering the obtained *p*, R, and F1 values, the Faster R-CNN model had the largest F1 value and the best overall evaluation in PyTorch, TensorFlow2, and Keras, respectively.

In terms of semantic segmentation network models, the U-Net model achieved the best overall evaluation in PyTorch and TensorFlow2, with the largest *p*, R, and AC values (89.11%, 90.28%, 98.37%, and 87.05%, 92.38%, and 98.28%, respectively). On the other hand, the PSPNet model had the best overall evaluation in Keras, with the largest *p*, R, and AC values of 88.36%, 71.55%, and 97.22%, respectively.

## 5. Conclusions

An experimental study of the best performance of bridge crack detection by coupling DLFs and CNNs was conducted using 2068 crack images based on three DLFs and five CNN models.

(1) The Faster R-CNN model showed the best detection effect among the object detection CNNs, which includes Faster R-CNN, SSD, and YOLO-v5(x). Similarly, among the semantic segmentation CNNs, the U-Net model exhibited the best segmentation effect compared to PSPNet.

(2) The detection efficiency of object detection and semantic segmentation CNN models varies under different DLFs. The Keras framework showed the best detection efficiency for the Faster R-CNN and SSD models, whereas the TensorFlow2 framework performed better for the PSPNet and YOLO-v5(x) models. Additionally, the PyTorch framework demonstrated the best detection efficiency for the U-Net model.

In short, the method and model of bridge crack detection with high precision and high performance can be obtained. For the object detection of bridge cracks, the Faster R-CNN model has the best performance in bridge crack detection under the Keras framework. For semantic segmentation of bridge cracks, the U-Net model has the best segmentation effect on bridge cracks under the PyTorch framework.

This paper presents an experimental study on the efficiency of object detection and semantic segmentation CNN models for bridge crack detection using PyTorch, TensorFlow2, and Keras. The results of this study are based on the image data set of bridge cracks in a simple background. The image data set of bridge cracks under complex interference background was not considered in the experiment, but the methods and results of this study can provide some practical basis for the research of bridge crack detection under complex interference background. The obtained results can serve as a significant reference for choosing the appropriate CNN model and DLF for bridge crack detection in future studies. Moreover, it establishes a solid theoretical and practical foundation for the development of real-time AI-based intelligent detection of building damage and unsupervised classification using remote sensing technology.

## Figures and Tables

**Table 1:** Comparisons among DLFs.

	Features	Release Date	Development and Maintenance	Core Languages	Interface Languages
Framework					
PyTorch		2017	Facebook, Twitter	C++, Python	Python
TensorFlow		2015	Google	C++, Python	C++, Python
Keras		2015	Google	Python	Python

**Table 2:** DLF configuration parameters.

	Framework	PyTorch	TensorFlow2	Keras
Configuration				
Python		3.6.13	3.6.13	3.6.15
scipy		1.2.1	1.4.1	1.2.1
numpy		1.17.0	1.18.4	1.17.0
matplotlib		3.1.2	3.2.1	3.1.2
opencv_python		4.1.2.30	4.2.0.34	4.1.2.30
torch		1.2.0	\	\
torchvision		0.4.0	\	\
tqdm		4.60.0	4.46.1	4.60.0
pillow		8.2.0	8.2.0	8.2.0
h5py		2.10.0	2.10.0	2.10.0
tensorflow_gpu		\	2.2.0	1.13.2
keras		\	\	2.1.5

**Table 3:** Detection results for different frameworks and networks in object detection.

	Faster R-CNN			SSD			YOLO-v5(x)		
	(a)	(b)	(c)	(a)	(b)	(c)	(a)	(b)	(c)
PyTorch	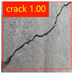	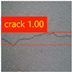	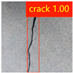	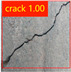	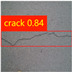	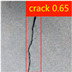	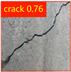	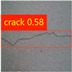	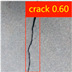
TensorFlow2	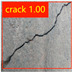	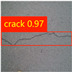	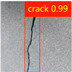	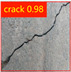	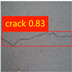	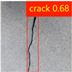	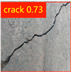	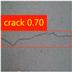	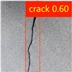
Keras	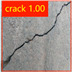	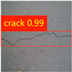	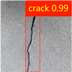	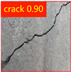	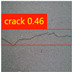	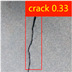	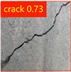	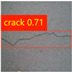	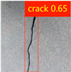

**Table 4:** Prediction confidence for different frameworks and networks in object detection.

	Faster R-CNN				SSD				YOLO-v5(x)			
	(a)	(b)	(c)	RMS	(a)	(b)	(c)	RMS	(a)	(b)	(c)	RMS
PyTorch	100%	100%	100%	0.000	100%	84%	65%	0.175	76%	58%	63%	0.093
TensorFlow2	100%	97%	99%	0.015	98%	83%	68%	0.150	73%	70%	60%	0.068
Keras	100%	99%	99%	0.006	90%	46%	63%	0.222	73%	71%	65%	0.042
Average	100%	99%	99%	\	96%	71%	65%	\	74%	66%	63%	\

**Table 5:** Mean and RMS values of confidence for different frameworks and networks in object detection.

	PyTorch		TensorFlow2		Keras	
	Mean	RMS	Mean	RMS	Mean	RMS
Faster R-CNN	0.993	0.011	0.978	0.019	0.964	0.122
SSD	0.782	0.275	0.731	0.214	0.765	0.200
YOLO-v5(x)	0.590	0.156	0.592	0.161	0.598	0.152

**Table 6:** Detection results for different frameworks and networks in semantic segmentation.

	U-Net			PSPNet		
PyTorch	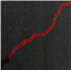	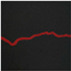	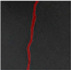	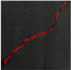	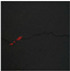	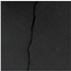
TensorFlow2	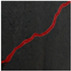	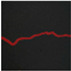	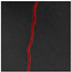	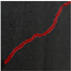	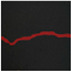	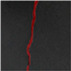
Keras	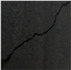	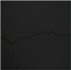	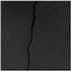	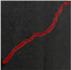	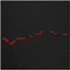	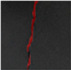

**Table 7:** Evaluation indicators for object detection models.

	Faster R-CNN			SSD			YOLO-v5(x)		
	PyTorch	TensorFlow2	Keras	PyTorch	TensorFlow2	Keras	PyTorch	TensorFlow2	Keras
*p* (%)	73.33	74.67	80.53	79.55	79.35	82.76	88.28	87.50	84.71
R (%)	64.71	67.06	71.37	54.90	51.37	56.47	50.20	54.90	52.16
F1	0.69	0.71	0.76	0.65	0.62	0.67	0.64	0.67	0.64

**Table 8:** Evaluation metrics for semantic segmentation models.

	U-Net			PSPNet		
	PyTorch	TensorFlow2	Keras	PyTorch	TensorFlow2	Keras
*p* (%)	89.11	87.05	47.97	84.38	85.62	88.36
R (%)	90.28	92.38	50.00	59.38	87.86	71.55
AC (%)	98.37	98.28	95.93	96.39	97.86	97.22

## Data Availability

The original data can be obtained from the open access online dataset by Li et al. [44].
